# Frequently rearranged and overexpressed δ-catenin is responsible for low sensitivity of prostate cancer cells to androgen receptor and β-catenin antagonists

**DOI:** 10.18632/oncotarget.25319

**Published:** 2018-05-11

**Authors:** Piyan Zhang, Janet Schaefer-Klein, John C. Cheville, George Vasmatzis, Irina V. Kovtun

**Affiliations:** ^1^ Center for Individualized Medicine, Mayo Clinic, Rochester, Minnesota, USA; ^2^ Department of Laboratory Medicine and Pathology, Mayo Clinic, Rochester, Minnesota, USA; ^3^ Molecular Medicine and Mayo Clinic, Rochester, Minnesota, USA; ^4^ Molecular Pharmacology and Experimental Therapeutics, Mayo Clinic, Rochester, Minnesota, USA

**Keywords:** delta catenin, prostate cancer, treatment, disease progression

## Abstract

The mechanism of prostate cancer (PCa) progression towards the hormone refractory state remains poorly understood. Treatment options for such patients are limited and present a major clinical challenge. Previously, δ-catenin was reported to promote PCa cell growth *in vitro* and its increased level is associated with PCa progression *in vivo.* In this study we show that re-arrangements at Catenin Delta 2 (CTNND2) locus, including gene duplications, are very common in clinically significant PCa and may underlie δ-catenin overexpression. We find that δ-catenin in PCa cells exists in a complex with E-cadherin, p120, and α- and β-catenin. Increased expression of δ-catenin leads to its further stabilization as well as upregulation and stabilization of its binding partners. Resistant to degradation and overexpressed δ-catenin isoform activates Wnt signaling pathway by increasing the level of nuclear β-catenin and subsequent stimulation of Tcf/Lef transcription targets. Evaluation of responses to treatments, with androgen receptor (AR) antagonist and β-catenin inhibitors revealed that cells with high levels of δ-catenin are more resistant to killing with single agent treatment than matched control cells. We show that combination treatment targeting both AR and β-catenin networks is more effective in suppressing tumor growth than targeting a single network. In conclusion, targeting clinically significant PCa with high levels of δ–catenin with anti-androgen and anti β-catenin combination therapy may prevent progression of the disease to a castration-resistant state and, thus, represents a promising therapeutic strategy.

## INTRODUCTION

A member of the p120-catenin subfamily, δ-catenin was originally discovered as a neuron-specific protein and later was shown to also play an important role in cancer. Studies report that δ-catenin is up-regulated in various human carcinomas including breast, lung, prostate, esophagus and colorectal cancer [1–4]. In a number of tumors, overexpression of δ-catenin is known to associate with poor prognosis [4–6]. For example, frequently observed in colorectal cancer, overexpression of δ-catenin was associated with higher grade and stage tumors and presence of metastases [4]. In esophageal squamous cell carcinoma, high expression of δ-catenin was noted to correlate with shorter survival [6]. Likewise, in non-small lung cancer, overexpression (compared to normal tissue) of δ-catenin was significantly associated with histological type, stage, differentiation, lymph node metastasis and a poorer survival [5].

Elevated expression of δ-catenin may be due to gene amplification, transcriptional activation and mutations in non-coding regulatory region [7, 8]. Mutations that can lead to changes in function of δ-catenin have also been identified. Skin, large intestine, stomach and lung cancers were among those cancers where point mutations were observed [9]. In a recent study, ectopic overexpression of δ-catenin protein in PCa cell lines was shown to induce mutagenesis leading to mutations impacting cellular function of δ-catenin [10]. In a functional study a number of these mutations were shown to promote tumor development in mouse model for PCa driven by expression of MYC gene. Mutated δ-catenin was shown to promote β-catenin translocation to the nucleus [10].

It is well established that δ-catenin can promote signaling through a canonical Wnt/β-catenin pathway activating LEF1-mediated transcription. One report noted that in PCa cells, δ-catenin interacts with E-cadherin in a competitive manner with p120 [11]. Each protein when bound to E-cadherin affected the stability of the other, thus modulating downstream signaling to β-catenin. δ-Catenin was shown to induce cleavage of E-cadherin by recruiting various proteases and stimulate a multi-layer growth of prostate cells [12]. By promoting E-cadherin processing, δ-catenin activated β-catenin-mediated oncogenic signals. When released from cadherins, δ-catenin can interact with actin network proteins, specifically with Rho family small GTPases. Increase in δ-catenin level led to decrease in binding between p190RhoGEF and RhoA, thus, significantly lowering levels of GTP-RhoA in the cell [13, 14]. These effects were reminiscent of RhoA inhibition [15]. In lung cancer cells, δ-catenin binds E-cadherin non-competitively, independent of p120, to regulate activity of small GTPases and cell cycle to promote malignant phenotype [15]. In addition to canonical Wnt pathway, δ-catenin acts in non-canonical Wnt pathway modulating transcriptional repressor Kaiso [16, 17].

A number of post-translational modifications of δ-catenin that affect its stability, function and subsequent downstream effects have been described [6, 13, 18]. Glycogen synthase kinase-3 GSK-3 was shown to phosphorylate δ-catenin targeting it for proteasome degradation [19]. As a part of GSK-3 signaling complex, δ-catenin is reported to promote β-catenin turnover [18]. E3 ligase β-TrCP-1 was identified as E3 ligase mediating ubiquitination of δ-catenin [20]. The latter study also reported that δ-catenin can be degraded through lysosome-dependent pathway. The degradation of δ-catenin was suggested to be inefficient when the protein is overexpressed [20]. Similar to p120 [21], δ-catenin is phosphorylated by Src kinase at multiple tyrosine residues [22]. Src-mediated phosphorylation enhanced ability of δ-catenin to induce translocation of β-catenin to the nucleus [22]. Furthermore, Src kinase activity is also essential for E3-ligase Hakai to stabilize δ-catenin. By stabilizing Src and subsequent inhibition of binding between δ-catenin and GSK-3β Hakai was shown to reduce ubiquitination and proteosomal degradation of δ-catenin [23].

The status of δ-catenin and its function in PCa have been studied extensively. Its overexpression is well documented [2, 24, 25], and has been found in PCa at both mRNA and protein levels [2, 25]. Extracellular accumulation of δ-catenin has also been reported [24]. In prostate cell lines overexpressing the protein, it was detected in the culture medium. Likewise, in cancer patients δ-catenin is accumulated in stroma, and its level is increased in the urine [24]. These findings suggested that δ-catenin could serve as biomarker for PCa and its progression. It was shown to promote PCa cell growth by altering the cell cycle and profiles of survival genes [26] and to activate the β-catenin signaling pathway [12]. In addition, δ-catenin was also reported to promote angiogenesis through stabilizing HIF-1α and activation of VEGF in the CWR22Rv-1 PCa cell line [27]. Together, these studies provided strong evidence in support of a significant role of δ-catenin in PCa progression. The ability of δ-catenin to activate Wnt/β-catenin signaling pathway in PCa poses a question whether β-catenin is a potential therapeutic target for PCa.

In this study, by analyzing the landscape of structural DNA alterations in a large series of PCa treated by radical prostatectomy we discovered that δ-catenin is frequently re-arranged in cases with clinically significant disease. The re-arrangements mostly represented duplications that are likely to account for higher expression levels of the protein in those cases. In a cell model overexpressing δ-catenin we found that it stimulates levels of its binding partners E-cadherin, p120, α and β-catenin and downstream targets of Tcf/Lef transcription factor. In LNCaP cells ectopically expressing δ-catenin, only the higher molecular weight form of δ-catenin protein was detected. The form was very stable and was not a product of phosphorylation, sumoylation or ubiquitination. When treated with inhibitors of β-catenin, overexpressing δ-catenin clones showed improved survival relative to parental cells. A combination treatment with anti-androgen and β-catenin inhibitor resulted in more efficient killing of cells with high levels of δ -catenin than either agent alone. Collectively our data suggest that PCa cells with overstimulated δ–catenin/β-catenin network are resistant to androgen deprivation and β-catenin inhibition. Combination therapy targeting both pathways simultaneously may be considered for PCa with high levels of δ-catenin.

## RESULTS

### Catenin Delta 2 gene (CTNND2) is frequently rearranged in prostate cancer

**Figure 1:**
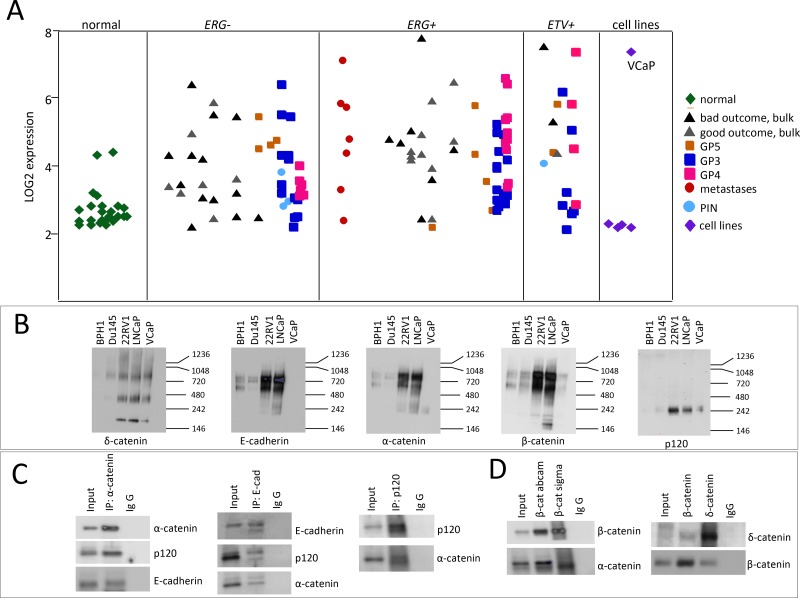
Characterization of expression of δ-catenin and its binding partners in PCa cases and cell lines (**A**) Graphical representation of CTNND2 expression in PCa cases based on microarray analysis [28]. Green corresponds to normal prostate epithelial cells, light blue is prostatic intraepithelial neoplasia (PIN), dark blue is Gleason pattern (GP) 3, magenta is GP4, orange is GP5, red corresponds to metastases, cell lines are shown in purple. Grey and black are bulk tissue (without laser capture microdissection) with good and bad (systemic progression) outcome respectively. Tumors are grouped into lacking TMPRSS-ERG fusion gene (ERG-), harboring TMPRSS-ERG fusion gene (ERG+) and harboring ETV fusion gene (ETV+). (**B**) Comparison of catenin binding complexes between different PCa cell lines. Cell lysates (50 μg) from BPH1, Du145, 22RV1, LNCaP and VCaP were subjected to nondenaturing gel electrophoresis and probed with indicated antibodies. (**C** and **D**). Characterization of binding partners of δ-catenin and β-catenin in LNCaP cells. Protein (300 μg) isolated from LNCaP cells was subjected to immunoprecipitation using IgG, anti-α-catenin, anti-β-catenin, anti-E-cadherin, anti-p120 or anti-δ-catenin antibodies immobilized on beads. The immunoprecipitates were resolved on SDS-PAGE electrophoresis and blotted with the indicated antibodies.

Overexpression of δ-catenin is found in PCa of Gleason score (GS) 7 and higher [2, 24, 25]. We profiled a large set of prostate tumors using gene expression microarray analyses [28]. RNA was extracted from laser capture micro-dissected tumors, different Gleason pattern (GP3, GP4 and GP5) were collected and analyzed separately. We observed higher levels of δ-catenin in GP4 (bright magenta) and GP5 (orange) compared to normal prostate epithelial cells (green, Figure 1A). The difference was particularly pronounced in tumors that had the TMPRSS2-ERG fusion. Consistent with the previous studies, we also found higher levels of δ-catenin in tumors of men that had recurrence (in black) as compared to the cases with no recurrence (in grey) (Figure 1A).

To gain an insight into potential cause of overexpression of δ-catenin we next compared CTNND2 gene status in surgically treated clinically significant and clinically insignificant PCa using mate pair whole genome sequencing [29, 30]. Clinically insignificant cases were defined as confined GS6 tumors, with volume <0.6 cm3; clinically significant group was comprised of tumors of higher grade (GS7 and higher). We found that the CTNND2 gene was frequently re-arranged in clinically significant prostate tumors, while insignificant cases did not harbor structural variations at the CTNND2 gene locus. Most of rearrangements in significant cases represented duplications (Supplementary Figure 1A–1D). In addition, cases of high GS (8 and higher) exhibited gains at chromosome 5 that involved CTNND2 gene (Supplementary Figure 1E), thus suggesting DNA alterations and copy number changes might be responsible for overexpression of δ-catenin in a subset of PCa. The fact that rearrangements at CTNND2 were only observed in clinically significant cases, and overexpression of δ-catenin is associated with worse prognosis strongly suggest that δ-catenin is a driver in PCa that promotes tumor progression by altering the cell cycle and stimulating cell growth of PCa [26].

### δ-Catenin induces levels of other catenin proteins in PCa cells

**Figure 2:**
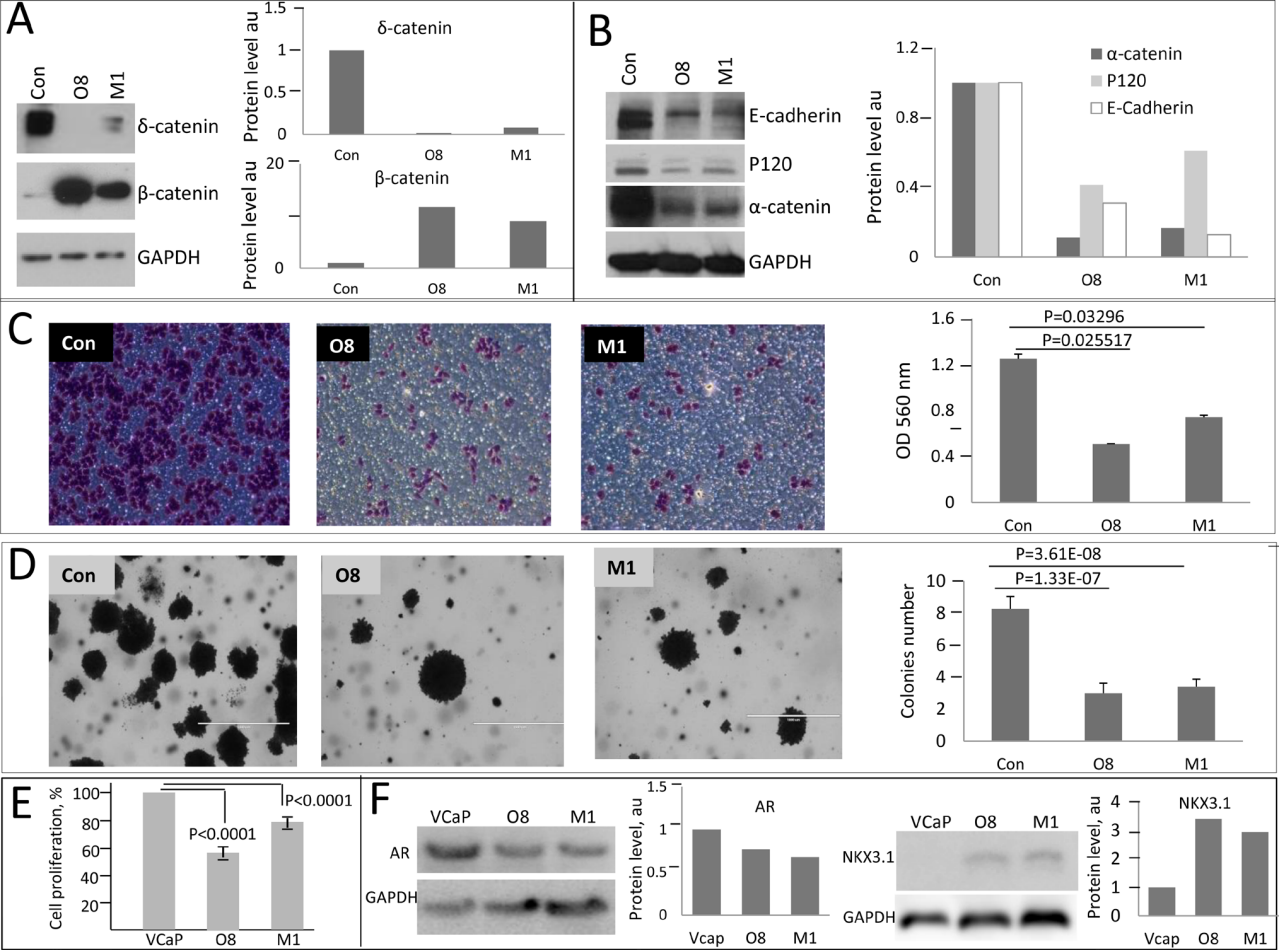
Oncogenic properties of PCa cells diminish upon knockdown of δ-catenin (**A**) SDA-PAGE and Western blot analysis of δ-catenin and β-catenin levels in clones (designated O8 and M1) with targeted CTNND2 gene. (**B**) Western blot analysis of indicated proteins in O8 and M1 clones. 30 μg of cell lysates were used in A and B, representative gels and corresponding quantification are shown. (**C**) Changes in cell migration were examined using Boyden chamber assay. Shown are images of stained cells with invasive phenotype after 36 hours. OD is optic density in arbitrary units measured at 36 hr. (**D**) Soft agar colony formation assay. Images of representative cells (left panel) and quantification (right panel) are shown. Data are presented as mean ± SD, based on 3 independent experiments. *P* values are as indicated. Quantification of expression of catenin proteins normalized to GAPDH expression is shown. Con is a vector only transfected control. (**E**) Proliferation of VCaP clones with knocked down expression of δ-catenin compared to parental cells at 48 hours after plating. Data presented as per cent of control parental VCaP cells. Mean and ±SD are shown based on 3 independent experiments, *p* values are as indicated. (**F**) SDA-PAGE and Western blot analysis of AR and NKX3.1 levels in VCaP clones and their corresponding quantification.

In order to elucidate perturbations at Wnt- β catenin signaling pathway due to overexpression of δ-catenin and examine their contribution to treatment sensitivity we generated a number of cell models with altered levels of δ-catenin. We first compared protein complexes involving key catenin proteins in a panel of PCa cell lines using non-denaturing gel electrophoresis (Figure 1B). The banding pattern for δ-catenin was similar between the examined cell lines, with the exception of BPH1, a benign hyperplasia cell line, in which very little to none of δ-catenin protein was detected (Figure 1B). LNCaP and 22RV1 cells showed nearly identical patterns for all catenin proteins and E-cadherin. In contrast, VCaP cells differed from all the other cell lines. Surprisingly, no E-cadherin and very little of α-catenin was detected in these cells (Figure 1B) suggesting that cadherin-catenin network in VCaP cells is altered. We next examined which proteins interact with δ-catenin in LNCaP cells by performing immunoprecipitation (IP) experiments. We observed that E-cadherin, p120 and α-catenin exist in one complex as each corresponding antibody pulled down the other two proteins (Figure 1C). β—Catenin was also found to bind p120, α-catenin and δ-catenin in LNCaP cells (Figure 1C, 1D). Since VCaP cells differed in binding pattern of catenin proteins, and very little of α-catenin and E-cadherin was detected using non-denaturing conditions (Figure 1B), we examined the effect of δ-catenin level alteration in these cells. Clones with knock-down levels of δ-catenin were generated (Figure 2A, Supplementary Figure 2) and characterized. The level of β-catenin in these clones was elevated compared to parental VCaP cells, while levels of E-cadherin, p120 and α-catenin were significantly diminished, along with a decrease in δ-catenin (Figure 2A, 2B). The levels of E-cadherin, p120 and α-catenin in parental VCaP cells as detected under denaturing conditions (Figure 2A, 2B) appeared relatively high compared to non-denaturing conditions (Figure 1B). The discrepancy can be explained by inaccessibility of binding sites for antibodies due to their occupation by binding partners in the latter case. Decrease in the level of δ-catenin led to decrease in tumorigenic properties of VCaP cells. Both clones O8 and M1 demonstrated lesser ability to migrate (Figure 2C) and form colonies in soft agar (Figure 2D) as well as decrease in proliferation rates (Figure 2E) as compared to parental VCaP cells. The result is surprising as the level of β-catenin, a protein considered to be a mediator of oncogenic properties, was increased. The morphology of cells with knock down δ-catenin differed from that of vector-transfected controls (Supplementary Figure 2B). The level of AR in O8 and M1 clones was diminished compared to parental cells (Figure 2F) despite the fact the β-catenin is reported to bind AR and stimulate transcription of androgen-dependent genes [31] including AR itself [32].

In contrast, another androgen-dependent gene, NKX3.1, was upregulated in δ-catenin knocked down clones (Figure 2F). This elevation is consistent with overall decrease in oncogenic abilities of these clones, as NKX3 is known to act in prostate cells as a tumor suppressor and is frequently lost during tumor progression [33–35]. Together these findings suggest that oncogenic action of δ-catenin in VCaP is not mediated by β-catenin.

**Figure 3:**
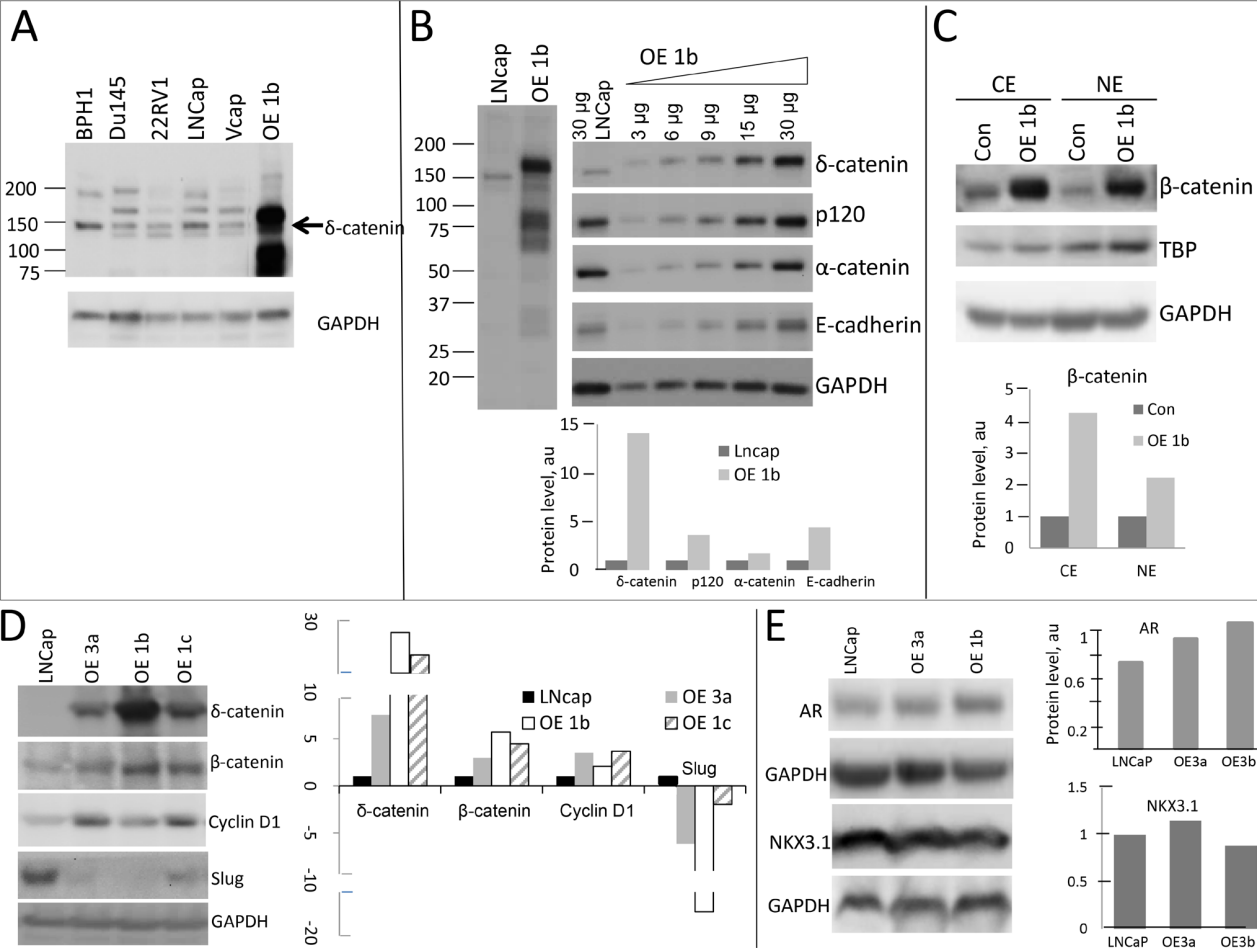
Activation of β-catenin pathway in LNCaP cells overexpressing δ-catenin (**A**) Comparison of levels of δ-catenin in overexpressing LNCaP clones and other PCa cell lines. Full length protein is depicted by arrow. (**B**) Levels of catenin proteins in δ-catenin overexpressing LNCaP clone (designated as OE1b) are shown. Gradient loading of total protein for OE1b clone was used to illustrate an increase in expression of each protein (right panel). Quantification graphs showing protein levels normalized to GAPDH level (arbitrary units, a.u.) are at the bottom. (**C**) Characterization of nuclear (NE) and cytoplasmic (CE) levels of β-catenin. Nuclear and cytoplasmic protein was isolated, β-catenin was detected by Western blotting using specific antibody. TBP and GAPDH were used as loading control of nuclear and cytoplasmic protein respectively. Normalized level of β-catenin in each compartment is shown at the bottom. (**D** and **E**) Characterization of levels of proteins, downstream targets of Wnt/β-catenin pathway (D), and androgen regulated genes AR and NKX3.1 (E) by Western blotting. Corresponding quantification is shown. Three clones (OE3a, OR1b, OE1c) overexpressing δ-catenin at various levels were examined. Total amount of 30ug of protein was used in each experiment, unless otherwise specified (in B). SDS-PAGE and Western blot conditions as in Figure 2.

LNCaP cells, compared to other PCa cell lines, showed relatively high expression of every protein involved in catenin network (Supplementary Figure 3), consistent with pattern in PCa samples. We, therefore, generated LNCaP clones expressing high levels of δ-catenin to explore a reverse scenario. Clone OE1b showed expression of δ-catenin more than ten times higher than parental cells (Figure 3A, 3B). Concomitantly, the levels of p120, E-cadherin and β-catenin also substantially increased (Figure 3B, 3C), likely due to an increase in their stability.

β-Catenin, when activated is known to translocate from cytoplasm to the nucleus to facilitate transcription of target genes [36–39]. To examine if this is the case in clones overexpressing δ-catenin, levels of β-catenin in both cellular compartments were compared to those of parental LNCaP cells. In OE1b clone, significantly higher levels of β-catenin in both cytoplasm and nucleus were observed (Figure 3C), supporting the notion that that δ-catenin directly or indirectly induces β-catenin which, in turn, can stimulate transcription of downstream genes. Consistent with that, we observed an increase in cyclin D1 levels a known downstream target for the β-catenin-Tcf/Lef transcription complex (Figure 3D). The level of AR was also slightly elevated in overexpressing clones (Figure 3E). Unlike VCaP (Figure 2F), LNCaP cells express a lot of NKX3.1 transcription factor, the level of which did not change significantly in the clones overexpressing δ-catenin (Figure 3E). The difference in regulation might be due to expression TMPRSS2-ERG fusion proteinδ-catenin, levels of β-catenin in both cellular compartments were compared to those of parental LNCaP cells. In OE1b clone, significantly higher levels of β-catenin in both cytoplasm and nucleus were observed (Figure 3C), supporting the notion that that δ-catenin directly or indirectly induces β-catenin which, in turn, can stimulate transcription of downstream genes. Consistent with that, we observed an increase in cyclin D1 levels a known downstream target for the β-catenin-Tcf/Lef transcription complex (Figure 3D). The level of AR was also slightly elevated in overexpressing clones (Figure 3E). Unlike VCaP (Figure 2F), LNCaP cells express a lot of NKX3.1 transcription factor, the level of which did not change significantly in the clones overexpressing δ-catenin (Figure 3E). The difference in regulation might be due to expression TMPRSS2-ERG fusion protein by VCaP cells. The level of Slug, a protein known to control epithelial to mesenchymal transition [40, 41], was diminished in LNCaP clones overexpressing δ-catenin (Figure 3D).

**Figure 4:**
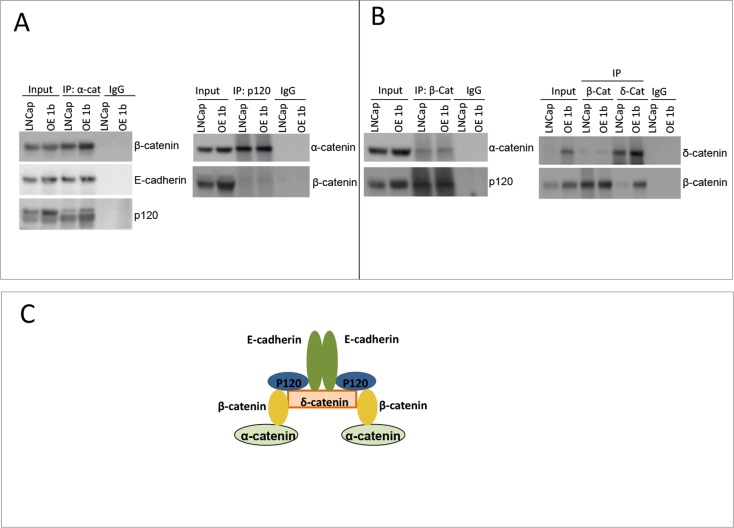
Characterization of δ-catenin binding complex (**A**, **B**) Comparison of binding partners of δ-catenin and β-catenin in LNCaP parental cells and overexpressing δ-catenin clone. Immunoprecipitation experiments were done as in Figure 1C. (**C**) A cartoon depicts binding complex of catenin proteins and E-cadherin in LNCaP cells, proposed on pull down experiments.

We next examined whether higher levels of δ-catenin in over-expressing clones affect the composition of the catenin binding complex. By a pull down assay using antibody to each protein in the putative complex as a bait, we found that E-cadherin, α-catenin, p120, δ-catenin and β-catenin exist in one complex (Figure 4A, 4B). There was no difference observed between parental LNCaP line and OE1b clone in the binding pattern of these proteins, compared in semi-quantitative manner. Although, the levels of these proteins were significantly higher in OE1b cells (Figure 3) no obvious discrepancy in the stoichiometry of binding partners was observed (Figure 4A). The levels of cross-precipitated β-catenin and δ-catenin, respectively were much higher in OE1b cells compared to parental LNCaP, suggesting that affinity of both proteins for binding each other does not change with an increase in δ-catenin levels.

Western blot analysis of cells expressing exogenous δ-catenin not only revealed its elevated levels but also pointed to a major product of a higher molecular weight than that in parental LNCaP cells (Figure 3B, Supplementary Figure 4A, 4B). Surprisingly, this endogenous form in LNCaP cells was not observed in OE1b clone at all. To test whether this was due to induction or/and stabilization of post-translationally modified δ-catenin we first examined and compared the phosphorylation level of δ-catenin in LNCaP parental and OE1b cells. Treatment of cell extracts with phosphatase did not result in changing protein migration, indicating that higher molecular weight form is not due to an increase in phosphorylation (Supplementary Figure 4A). Likewise, no changes in migration were observed for α-catenin, p120 and E-cadherin (Supplementary Figure 4B).

Ubiquitination is an important step in the process of targeting proteins for proteosomal degradation [42] and was shown to play a role in controlling β-catenin [43, 44] and δ-catenin stability [20]. In addition, monoubiquitination is involved in non-degradative activities and is shown to impact many biological processes such as the regulation of gene transcription, protein trafficking, and DNA repair [45, 46]. We, therefore, next tested whether treatment with ALLN, calpain inhibitor I, which is known to block the proteasome pathway [47, 48] changes the levels of δ-catenin in LNCaP parental cells and clones overexpressing δ-catenin (Supplementary Figure 4C, left panel). Surprisingly, treatment with ALLN did not result in increase of δ-catenin stability in LNCaP parental cells, as the protein levels between treated and untreated cells did not differ. Likewise, no changes in levels of δ-catenin were observed in overexpressing clone OE1b (Supplementary Figure 4C). This is in contradiction with earlier reports showing increase in δ-catenin level upon stabilization caused by proteasome inhibition [20, 23].

Among other protein modifications, sumoylation is known to affect localization, transcriptional regulation and enzymatic activity [49]. It was also shown to be involved in cellular stress response, playing important roles in recovery from heat shock ischemia and surviving the stress of tumorigenesis [50, 51]. Using sumoylation inhibitor 2-DO [52] we examined whether the migration of the overexpressed form of δ-catenin in OE1b clone changes following treatment. No changes were observed, suggesting that the higher molecular weight form of δ-catenin was not due to its sumoylation (Supplementary Figure 4C, right panel).

### Targeting β-catenin in cells with altered levels of δ-catenin

**Figure 5:**
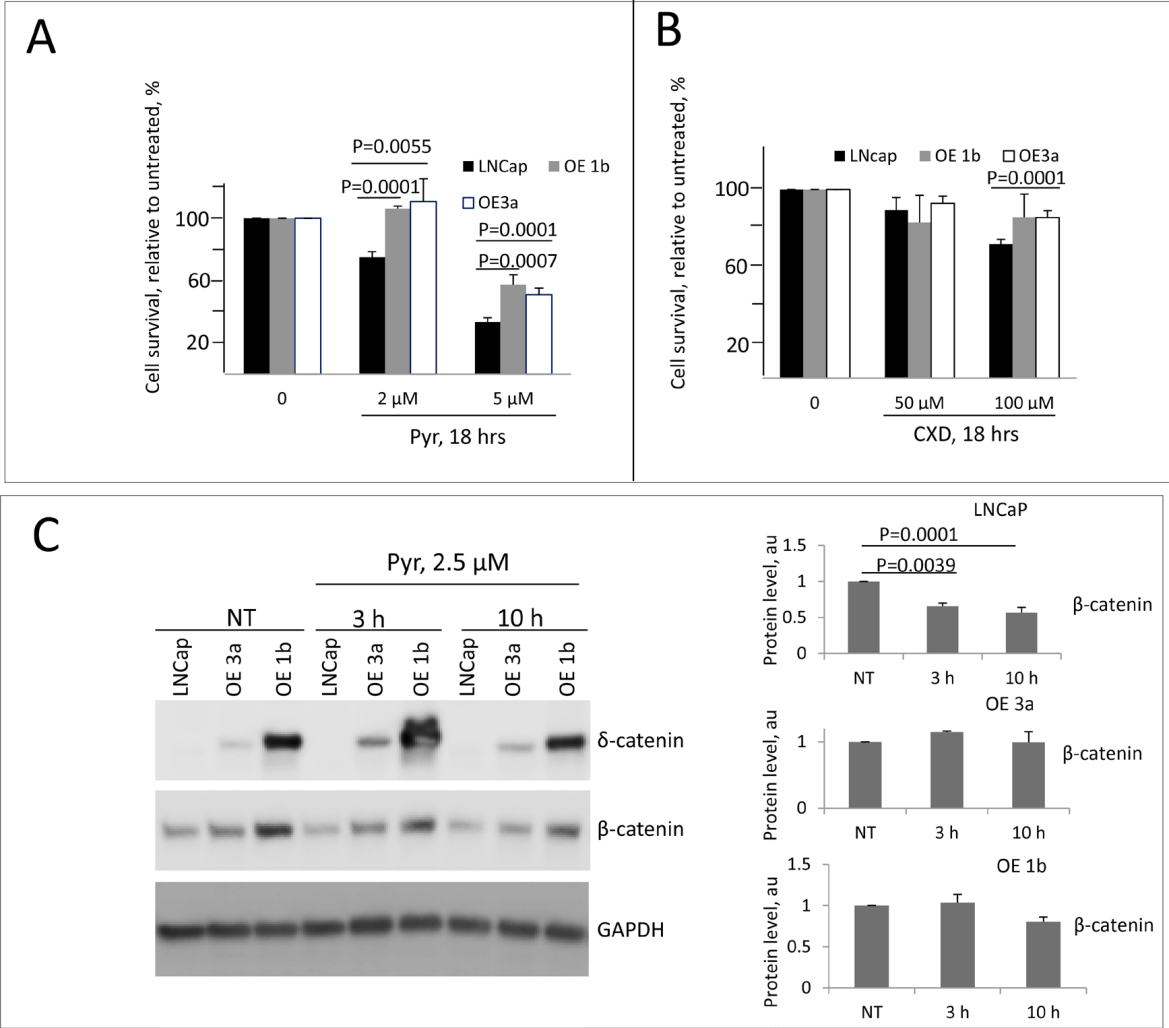
Sensitivity of δ-catenin overexpressing cells to treatment with β-catenin inhibitor pyrvinium (**A, B**) LNCaP, OE3a and OE1b cells were grown in RPMI 1640 supplemented with 10% charcoal stripped FBS for 48 hours and then treated with pyrvinium (Pyr, in A.) or casodex (CXD, in B). Concentrations and time are as indicated. Data are presented as mean ± SD, based on 3 independent experiments. (**C**) Western blotting showing changes in levels of δ-catenin and β-catenin upon indicated treatment; quantification is shown in right panel.

**Figure 6:**
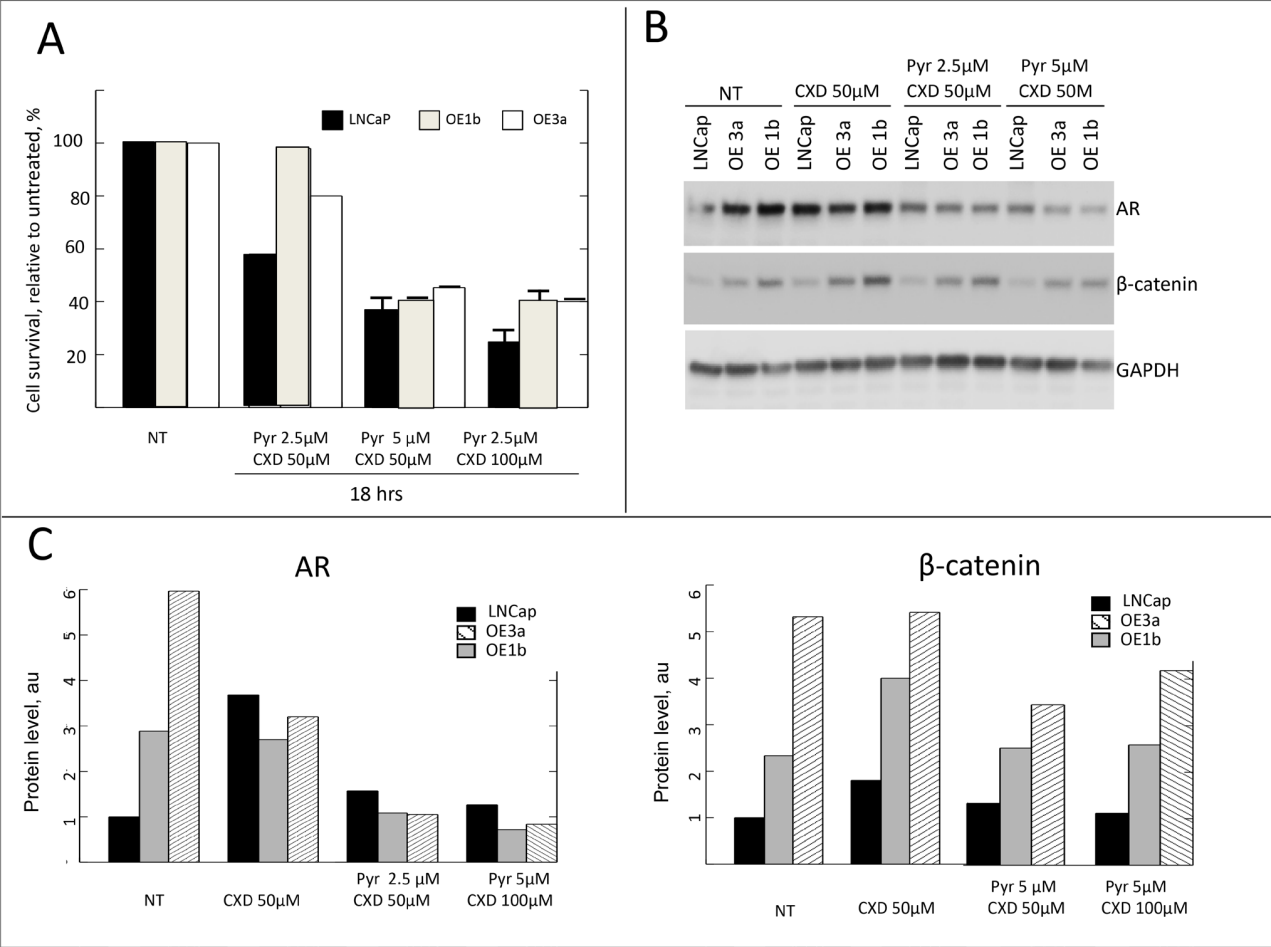
Sensitivity of δ-catenin overexpressing cells to combination treatment with β-catenin inhibitor and anti-androgen (**A**) Survival of indicated clones is expressed as % of that of corresponding untreated clones. Data are presented as mean ± SD, based on 3 independent experiments. (**B**) Western blot showing changes in levels of AR and β-catenin upon indicated treatment, quantification is shown in (**C**).

The significance of Wnt/β-catenin pathway in PCa is well documented [53–57], and its inhibition has anti-tumor effects in several experimental models [58–61]. Moreover, interplay of the β-catenin pathway and androgen receptor (AR) signaling pathway in driving PCa progression has also been reported. AR binds β-catenin directly to stimulate transcription of androgen-dependent genes [31] including AR itself [32]. Concordant re-activation of AR and Wnt signaling pathways accompanied by an increase in expression of both AR and β-catenin was reported in castration-resistant PCa [62]. The levels of β-catenin and its downstream targets are elevated in clones overexpressing δ-catenin (Figure 3B) thus suggesting activation of the pathway. We, therefore, tested the sensitivity of these clones to β-catenin inhibitors. Pyrvinium is known to potentiate casein kinase 1α activity thus promoting β-catenin degradation and inhibiting Wnt/β-catenin signaling and proliferation [63, 64]. Treatment of cells in culture with pyrvinium resulted in a reduction of both cytoplasmic and nuclear β-catenin [64]. After 18 hours of incubation with pyrvinium at 5 μM, all cells showed a significant reduction in survival (Figure 5A). Parental LNCaP cells were more sensitive than any of the clones overexpressing δ-catenin. A decrease in survival in parental cells, indeed, was observed even at a lower concentration (Figure 5A). This effect in parental cells was accompanied by significant decrease in the total level of the β catenin (Figure 5C), consistent with the reported mechanism of action of pyrvinium [64] and suggesting that inhibition of β-catenin pathway in LNCaP cells results in cell killing. Compared to parental LNCaP cells, clones over-expessing δ-catenin demonstrated milder response to pyrvinium. Only higher concentration of 5 μM caused a decrease in survival, and no significant reduction in level of β-catenin was observed (Figure 5A and 5C). This suggests that the pathway in cells overloaded with β-catenin cannot be efficiently blocked. Contrary to pyrvinium, there was no difference in response to CCT31374 [63] another inhibitor of β-catenin previously shown to inhibit cell growth, between parental cells and clones overexpressing δ-catenin (Supplementary Figure 5A). Viability of AR-dependent LNCaP cells, either parental or δ-catenin high expressing clones was not significantly altered by treatment with anti-androgen casodex upon 18 hours of incubation (Figure 5B). Only a slight decrease was noted in survival of parental cells at 100uM concentration. Because of cross-talk between of β-catenin and AR pathways where AR stimulates expression of β-catenin and vice versa, we next examined whether combined inhibition of these pathways would lead to more efficient cell killing. When responses between parental and overexpressing clones were compared, similarly to a single agent treatment, the combination was more effective in killing parental cells (Figure 6A). Only 20% of cells in LNCaP parental sample survived upon 18 hr incubation with pyrvinium and casodex, while up to 40% were still alive in δ-catenin clones. Although, not as sensitive to a combination treatment as parental cells, δ-catenin overexpressing cells showed greater response to dual inhibition than to either drug alone (Figure 5A, 5B and Figure 6A). Reduction in viability in δ-catenin clones was accompanied by a significant decrease in AR level but not β-catenin (Figure 6B, 6C). Since VCaP cells with δ-catenin knockdown also showed increase in β-catenin level (Figure 2A), we next examined sensitivity of these clones to the same treatments for comparison. Similar to LNCaP, VCaP parental cells were more sensitive to pyrvinium than their derivative O8 and M1 clones (Supplementary Figure 5B) expressing approximately 10 fold more of β-catenin (Figure 2A). Likewise, combination treatment with caxodex and pyrvinum together was more efficient in killing O8 and M1 cells. On the other hand, survival of parental VCaP cells upon combination treatment was not significantly different from that with pyrvinium alone (Supplementary Figure 5B).

Collectively, these results indicate that combination treatment targeting AR and β-catenin pathways in cells with high levels of β-catenin is more promising approach than androgen ablation alone.

## DISCUSSION

Progression of PCa to androgen-independent state, is a main cause of treatment resistance, tumor progression and PCa-related death. Castrate resistant PCa is associated with a poor prognosis and mean survival time of 16–18 months [65]. There are only a few treatment options in this setting, and they generally fail [66, 67]. Better therapies are needed to improve survival of these patients. The mechanism by which PCa progresses to castration-resistant disease is not well understood and remains a major clinical challenge. Although, an increase in androgen-independent signaling due to elevated AR receptor expression/gene amplification, AR gene mutations and its activation by other transcription factors is considered to be a major pathway underlying hormone resistance, other networks including Wnt/ β-catenin are implicated in the PCa progression [56, 68, 69]. For example, β-catenin levels were reported to correlate with high Gleason score, AR expression and hormone-refractory metastatic disease [53, 54]. In hormone resistant cases, AR and Wnt signaling pathways are activated, both proteins overexpressed [62] and co-localized in the nucleus [56]. Progression-driving potential of β-catenin in PCa was also established in mouse models *in vivo* [57, 70]. AR was shown to act through Wnt/β-catenin network in ligand independent manner [56]. Furthermore, β-catenin in PCa was demonstrated to promote epithelial-mesenchymal transition through regulation of HIF-1α [71, 72]. The major cause underlying β-catenin activation during PCa progression is believed to be loss or down-regulation of E-cadherin [55, 73–75]. A recent report showed another mechanism for β-catenin activation in castrate resistant PCa, and that is the expression of miR-744 which targets multiple negative regulators of Wnt/β-catenin signaling, including SFRP1, GSK3β, TLE3 and NKD1 [76]. Inverse correlation between survival of patients and expression of miR-744 was found, further implicating Wnt/β-catenin signaling in progression of PCa. In our study we found a novel mechanism for β-catenin pathway activation. We show that a member of p120-subfamily, δ-catenin, which is frequently overexpressed in PCa [2, 24, 25], can activate β-catenin signaling, increasing its levels and affecting oncogenic properties of PCa cells (Figure 3). Interestingly, we found that rearrangements and copy number changes at the CTNND2 locus are frequent but restricted to clinically significant PCa cases, supporting its role in disease progression. As most of the rearrangements are represented by gene duplications and copy number changes- by gains, this discovery points to an additional mechanism underlying an increase in δ-catenin expression. There are conflicting reports as to role of δ-catenin within the Wnt/β-catenin signaling pathway. Manipulating levels of δ-catenin, we created several different cell models that allow the tracking of key elements in the pathway and tested the sensitivity of these models to pathway specific treatments. Earlier studies suggested that δ-catenin may compete with p120 for E-cadherin binding [11]. Other reports showed a somewhat independent role of δ-catenin [3]. When we examined and compared δ-catenin binding complexes in a panel of PCa cell lines and benign BPH1 cells we found that the binding pattern for δ-catenin did not differ between cancer cell lines qualitatively. Lesser amount of protein was detected in DU145 cells. The binding pattern was distinctively different in benign BPH1 cells showing almost no δ-catenin. In contrast, very little E-cadherin was detected and diminished levels of α-catenin and β-catenin were observed in VCaP cells despite the same amount of starting cell material. This posed a question whether binding partners of δ-catenin in VCaP cells differ from those in other three PCa cell lines. Moreover, knockdown of δ-catenin in VCaP cells resulted in further reduction of levels E-cadherin, p120 and δ-catenin but surprisingly elevated β-catenin. This effect was accompanied by decrease in proliferation and ability of cells to invade and form colonies in soft agar (Figure 2C–2E). Collectively, observations in VCaP cells suggest that oncogenic effects of δ-catenin in these cells are likely not mediated by activation of β-catenin whose stability/levels increased upon knockdown of δ-catenin. Our data rather are consistent with the notion that in VCaP cells, which have much lower levels of p120, E-cadherin and α-catenin, δ-catenin is associated with other partners and, perhaps, knock down of δ-catenin results in increase of β-catenin stability as it becomes available for other binding partners. The difference in regulation of Wnt/ β-catenin pathways in these cells might be due to the presence and expression of the TMPRSS2-ERG fusion protein. It was reported that ERG transcription factor activates the Wnt/LEF1 signaling pathway by multiple mechanisms including binding to promoters of various Wnt genes, increasing level of active β-catenin level and directly stimulating LEF1 transcription [77].

The level of Slug, known to control epithelial to mesenchymal transition, was diminished in LNCaP clones overexpressing δ-catenin (Figure 3D). This is surprising since Lef-1, Tcf-1 and Tcf4 transcription factors along with β-catenin are known to bind Slug promoter *in vivo* to stimulate its expression [78], and levels of nuclear β-catenin were increased in cells overexpression δ-catenin (Figure 3C). On the other hand, levels of Slug are known to be maintained through ubiquitin-mediated proteasomal degradation [79] which is shown to be initiated by GSK3β-dependent phosphorylation [80–82]. Since stability of δ-catenin decreases its affinity to GSK3β, it is possible that release of GSK3β from δ-catenin complex leads to stimulation of Slug phosphorylation followed by ubiquitination and its subsequent degradation. Few reports suggest that function of Slug in PCa goes beyond its role in epithelial to mesenchymal transition. Studies demonstrated lower Slug expression in epithelial cells of PCa compared to their normal counterparts [83, 84]. The down-regulation observed in most epithelial cells, however, was not present in i) the cells of invasion front in high-grade PCa, ii) areas with neuroendocrine differentiation and iii) lymph node metastasis [85]. Earlier study compared Slug expression in a panel of PCa cell lines and reported elevated levels in LNCaP, PC-3 and 22RV1 [50]. Contrary to other reports, this study revealed that increase in expression of Slug decreased cyclin D1 level in PC-3 and DU-145 cells. Vice versa, ectopic expression of cyclin D1 showed Slug-mediated inhibition of proliferation of these cell lines [86]. Our results are also consistent with the notion that in PCa cells, Slug is a negative regulator of proliferation as we found that a decrease in its expression caused by δ-catenin led to overexpression of cyclin D.

An increase in β-catenin level was concomitant with overexpression of δ-catenin in LNCaP cells (Figures 3 and 4). The levels of all examined members of pathway were higher in clones overexpressing δ-catenin than parental LNCaP cells suggesting that their stability and/or transcriptional regulation was δ-catenin-dependent. Furthermore, co-immunoprecipitation experiments revealed that the binding pattern for members of the pathway was preserved as no differences were observed between parental and δ-catenin high expressing cells (Figure 4). Together, the findings indicate that there is no competition between δ-catenin and other catenins and E-cadherin in LNCaP cells. Interestingly, the overexpressed form of δ-catenin was higher in molecular weight than endogenous protein (Figure 3B, Supplementary Figure 4), and its stability was not dependent on phosphorylation, ubiquitination or sumoylation. In contrast to earlier studies that reported that phosphorylation by Src kinase stabilizes δ-catenin and enhances its ability induce translocation of β-catenin to the nucleus [22, 23], we found no evidence that the phosphorylation status altered levels of δ-catenin in LNCaP cells. Both parental and cells overexpressing δ-catenin maintained the same protein level after treatment with phosphatase. Moreover, the higher molecular weight form was the dominant species in the latter cells with no endogenous form detected. As no known protein modification that would alter either mobility or the level of δ-catenin in overexpressing cells was found, we speculate that exogenously over-expressed form of δ-catenin stimulates expression of different isoform of the protein or perhaps its stability, possibly through β-catenin transcription activation loop. The same mechanism can be responsible for maintenance of high levels of β-catenin itself.

Cooperation of AR network and β-catenin in regulating the growth of prostate cells is well established [56, 69], and β-catenin inhibition was shown to have anti-tumor effects [58, 59, 87]. In this study we find that targeting cells overexpressing δ-catenin or β-catenin with anti-β-catenin therapy is inefficient (Figure 5, Supplementary Figure 5). Compared to parental LNCaP and VCaP cell the derivative clones showed very little sensitivity to either pyrvinium or CCT031374. As both cytoplasmic and nuclear β-catenin levels were several fold higher in cells with altered δ-catenin expression, one can speculate that higher doses of β-catenin inhibitors would be required to overcome its growth stimulating effects. Alternatively, other pathways contributing to β-catenin stimulation may need to be considered for targeting.

In summary, our study reports on complexity of interaction between catenin proteins within Wnt signaling pathway and its cross-talk with AR network. We show that δ-catenin in PCa induces β-catenin which, in turn, impacts responses to existing androgen deprivation therapy and currently under development β-catenin inhibitors. Further studies are needed to refine therapeutic interventions in hormone refractory PCa driven by Wnt/β-catenin.

## MATERIALS AND METHODS

### Antibodies and reagents

The following antibodies were used for western blotting and co-immunoprecipitation: δ-catenin (Abnova, cat. H00001501-A01), β-catenin (Sigma, cat. c2206), p120 (Santa Cruz, cat. SC-13957), α-catenin (Novus, cat. NB100-74356), cyclin D1 (Cell Signaling, cat. 2922S), NKX3.1 (R&D systems; cat. AF6080) and Slug (Cell signaling; cat. 9585). Inhibitors pyrvinium (cat. P0027), CCT031374 (cat. SML1399), ALLN (cat. A6185), 2-D08 (cat.. SML1052) and casodex (cat. B9061) were purchased from Sigma. λ-PP (cat. P0753S) was from New England Biolabs.

### Cell culture and generation of stable clones

Prostate cell lines LNCap, BPH1, 22RV1, VCaP and DU145 were purchased from ATCC. LNCaP, BPH1 and 22RV1 cells were maintained in RPMI1640 with 2 mM L-glutamine and 10% fetal bovine serum in a CO2 (5%) incubator at 37°C. VCaP and DU145 cells were grown in DMEM medium supplemented with 10% fetal bovine serum. Stable LNCaP clones overexpressing δ-catenin were generated by transfection of CTNND2 Lentifect™ Lentiviral Particles (NM_001332.2, GeneCopoeia™), single clones were selected by addition of puromycin at final concentration of 2 μg/ml. Expression level was confirmed by Western blot analysis.

VCaP cells with knock down levels of δ-catenin were generated using CTNND2 gene specific knock out kit via CRISPR (KN224464, Origene) according manufacturer's instructions. TurboFectin (TF81001, Origene) was used to transfect gRNA vectors, scramble negative control and donor vector. The integration of functional cassette was confirmed by PCR using genomic DNA from selected puromycin-resistant clones and specific primers: CGAAGAGG TTCACTAGGCGCGCCTTTCT (forward) and CGAAGAGGTTCACTAGGCGCGCCTTTCT (reverse).

### Validation of rearrangements at CTNND2 locus

PCR was performed on DNA isolated from PCa samples [28] using rearrangement (breakpoint)-specific primers and HotStar Taq DNA Polymerase Kit (Qiagen), on a GeneAmp PCR System 9700 (Applied Biosystems). Amplification products were visualized on a 1% agarose gel, single bands were excised, DNA was extracted, and Sanger sequenced using the appropriate primers on a 3730 ×1 DNA Analyzer (Applied Biosystems). A mixed pool genomic DNA sample was used as a normal DNA control (Promega).

### Western blotting and immunoprecipitation

Cells for protein analysis were lysed in NTEN buffer (100 mM NaCl, 20 mM Tris.HCl pH8.0, 0.5 mM EDTA, 0.5% Nonidet P-40) supplemented with complete protease inhibitor cocktail (Roche, cat. 11836170001) and phosphatase inhibitor cocktail II (Boston BioProducts, cat. BP-480). Protein concentration was determined using Pierce BCA Protein Assay Kit (Thermo Scientific, cat. 23225). Protein was separated by SDS-PAGE, transferred to nitrocellulose membrane and blotted with corresponding antibodies. For pull down experiments cell lysates were incubated with precipitating antibodies immobilized on protein A/G PLUS-Agrose beads (Santa Cruz, cat. sc-2003) for 4-16 hours. Samples were washed with NTEN buffer containing protease inhibitor cocktail. Immunoprecipitates were separated by SDS-PAGE and transferred to nitrocellulose membranes for Western blotting.

Native gel electrophoresis was performed to detect protein complexes. Sample Prep Kit was used to extract protein and gel electrophoresis was done using NuPAGE Bis-Tris Gels (Thermo Fisher Scientific, cat. BN1002BOX) according to manufacturer's instructions.

### Cell migration, cell proliferation and colony formation assays

The cell migration assay was performed using polycarbonate membrane with 8 μm pore size (Cell Biolabs, cat. CAB-100). 2 × 10^5^ cells in the serum free medium were added to the inside of each membrane, FBS-containing medium was added to each matched well on 24-well plate. After 48 hour incubation, noninvasive cells were removed from the upper surface of the transwell membrane, and cells which migrated through the membrane were incubated with Cell Stain Solution, washed with Extraction Solution, absorbance at 560 nm was measured using GloMax-Multi Detection System (Promega).

For colony formation assay 5 × 10^3^ cells were seeded on 6-well soft agar plate, maintained at 37°C for 10–20 days, washed with PBS and stained with Giemsa solution. The number of colonies (containing >50 cells) was counted using the following formula: colony formation efficiency = number of colonies/number of cells seeded.

MTS cell proliferation assay was performed following manufacturer instructions (Promega Corp., cat G3581). Cells were plated in 96-well plates and absorbance values were obtained using a GloMax multi detection system (Promega).

### Drug treatment and cell proliferation assay

5 × 10^4^ cells were seeded in 24-well plate in growth medium and maintained for 48 hours. For treatments growth medium was replaced with RPMI1640 containing 10% charcoal stripped FBS (Gibco, cat. 12676-011) and cells were maintained at 37°C for 24 hours more. Pyrvinium and/or casodex were added at indicated concentrations and cells were incubated with drugs for designated time. Cell viability was measured using CellTiter 96 AQuious assay kit (Promega, cat. G5421) according manufacturer's instruction.

### Statistical analysis

All data were presented as mean ± SD. The mean was the average of at least triplicate samples in each experiment. Each experiment was repeated at least three times. Student's *t*-test was used to analyze the results. Differences were considered to be statistically significant at *p* < 0.05.

## SUPPLEMENTARY MATERIALS FIGURES


