# Supplementary material for: Frequently rearranged and overexpressed δ-catenin is responsible for low sensitivity of prostate cancer cells to androgen receptor and β-catenin antagonists
Source: Oncotarget. 2018 May 11;9(36):24428–42. doi: 10.18632/oncotarget.25319 (PMC5966253; doi:10.18632/oncotarget.25319)
Supplement: Supplementary file 1 [file oncotarget-09-24428-s001.pdf]

Frequently rearranged and overexpressed δ-catenin is responsible for low sensitivity of prostate cancer cells to androgen receptor and β-catenin antagonists

SUPPLEMENTARY MATERIALS

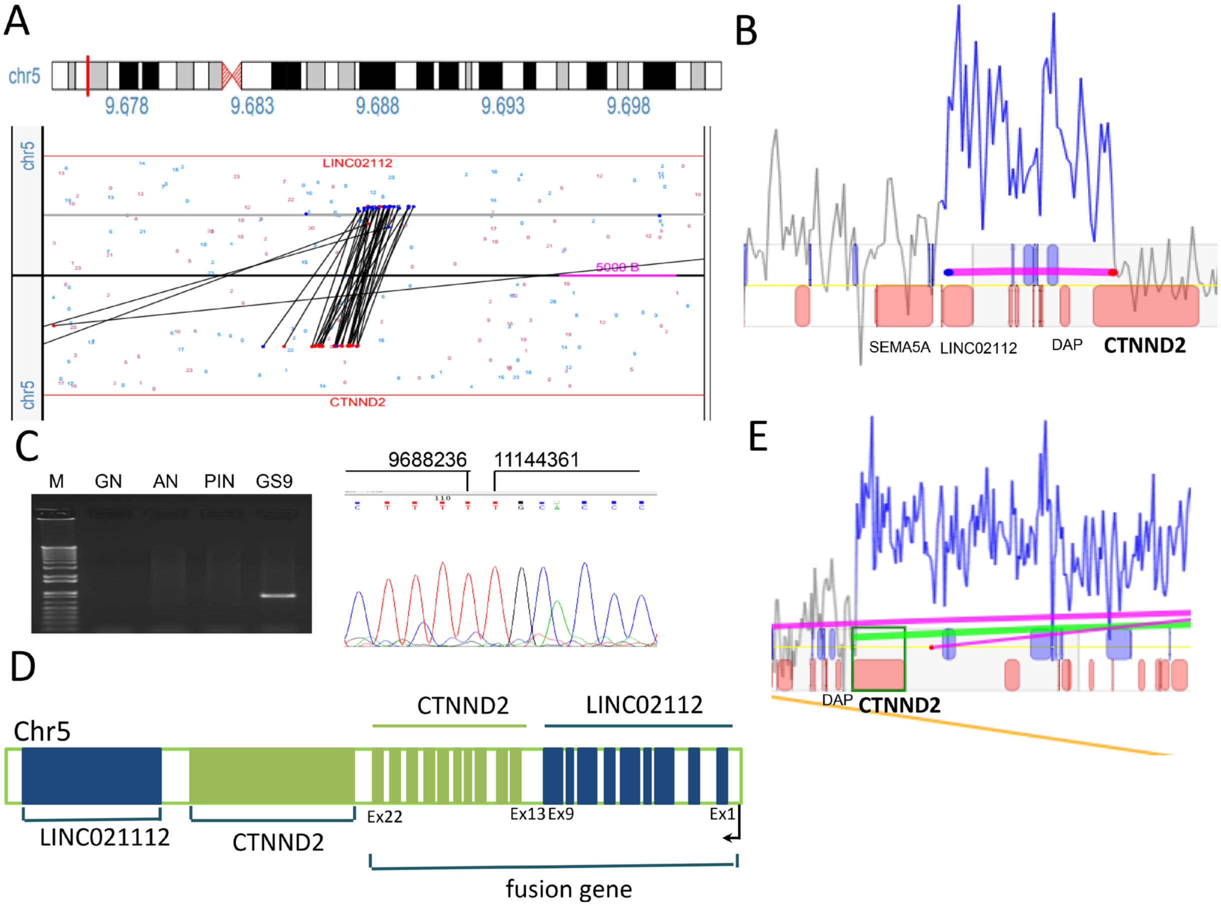

Supplementary Figure 1: (**A**–**C**) Validation analysis of duplication event at CTNND2 gene identified by mate pair sequencing [30, 88]. (A). A junction plot showing regions of chromosome 5 involved in the break. Vertical connecting lines represent aberrant read-pairs; blue dots refer to mapping to (+) strand, whereas red ends indicate mapping to (–) strand. Horizontal red line indicates that both genes are located on (–) strand. (B) A close-up segment of the genome plot illustrating alteration at chromosome 5 involving CTNND2 gene depicted in (A) Blue lines correspond gains, magenta line connects breakpoints at indicated genes that form an aberrant junction (associated reads are shown as vertical lines in A). Genes are shown as blocks, red and blue colors correspond to (–) and (+) strands respectively. (C) A presentative agarose gel image (on the left) showing PCR product amplified using specific to the rearrangement primers. N is normal genomic DNA, AN is adjacent histologically normal tissue, PIN is adjacent prostate intraepithelial neoplasia and GS9 is the tumor of the same PCa case. Chromatogram of sequence analysis of the breakpoint is shown on the right, the positions from reference genome are indicated (**D**). A schematic showing rearrangement at the CTNND2 locus as determined by mate-pair analysis. The arrow indicate transcription start site on (–) strain. Specific exons forming a fusion gene involving LINCO2112 and CTNND2 are indicated. (**E**) A close-up segment of the genome plot showing a gain (indicated by blue lines) at chromosome 5 involving CTNND2 locus (depicted in green box) in GP5 case of PCa.

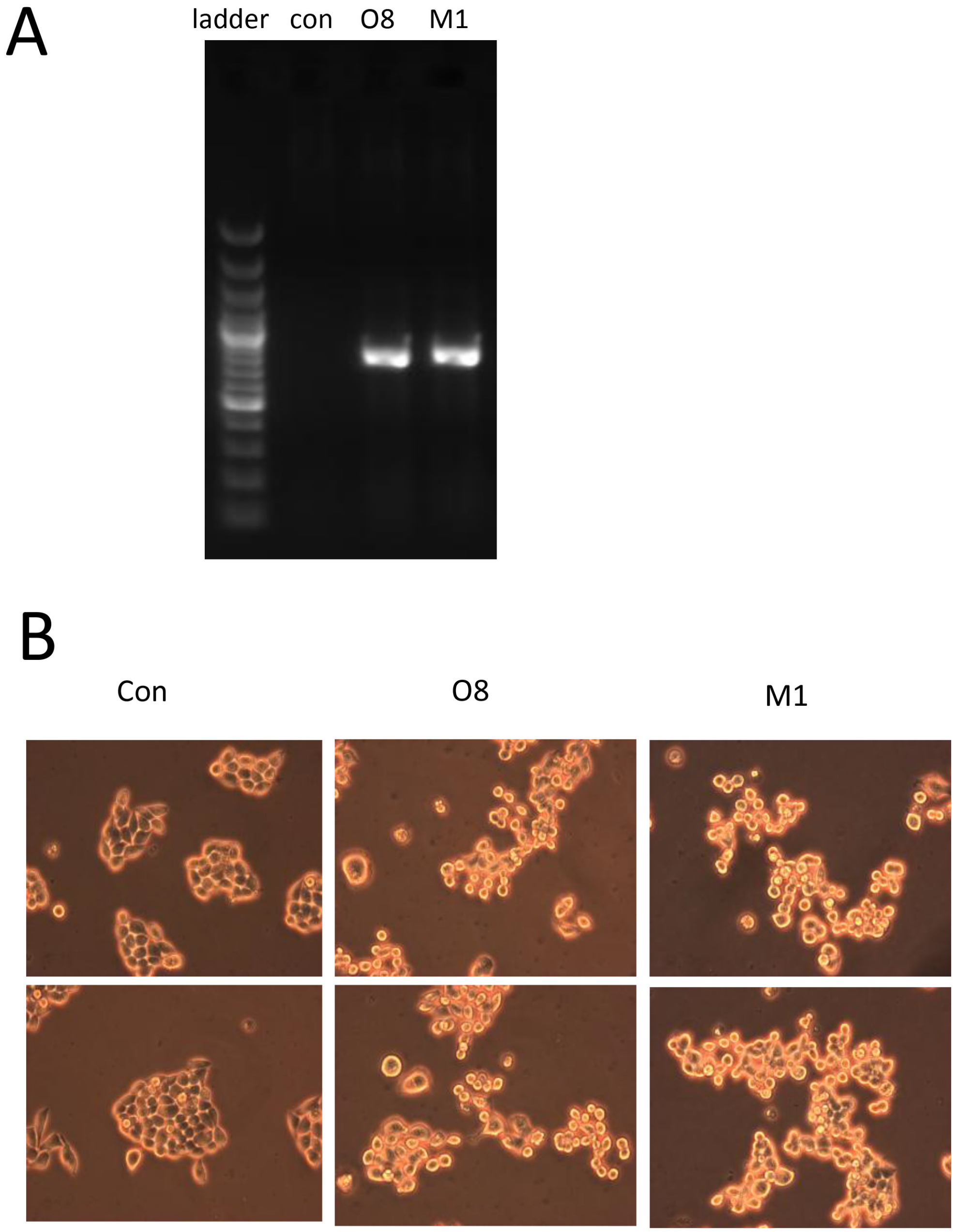

Supplementary Figure 2: Validation of targeting vector integration in CTNND2 knockdown clones O8 and M1. (**A**) Genomic DNA of puro-resistant O8, M1 clones and parental VCaP cells was used in PCR with forward primer (upstream of 5’ end of the left arm) and reverse primer (GFP region). (**B**) Light images (40×) of VCaP vector transfected (con) and VCaP clones (O8, M1) with knockdown δ-catenin, taken using Life EVOS FL Microscopy Cell Imaging System. 2×10^5 cells/well were seeded and were grown for 4 days until imaged.

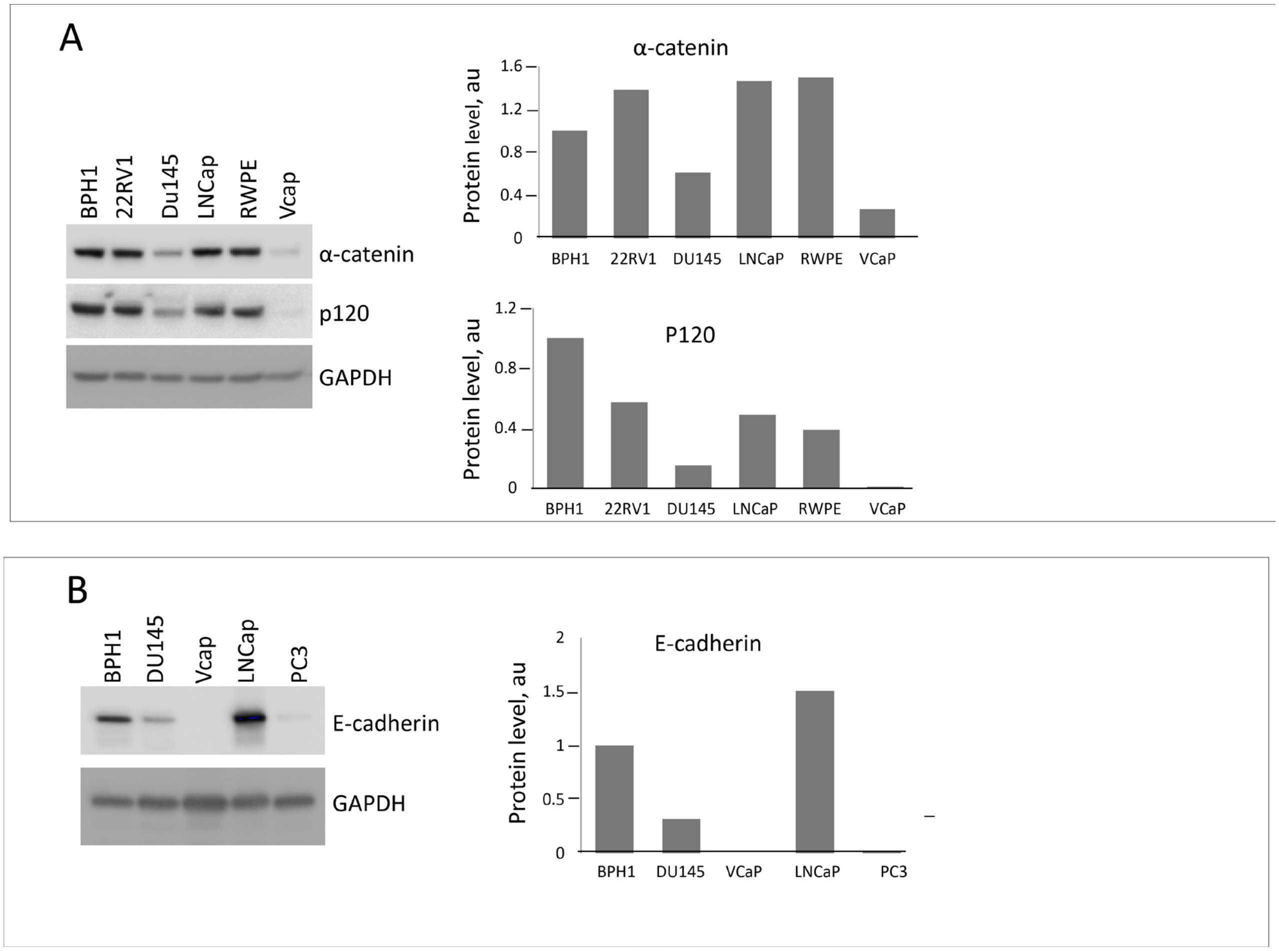

Supplementary Figure 3: Characterization of levels of catenins and E cadherin in additional PCa cell lines (**A**, **B**). Total 30 µg of protein was used in Western blot analysis. Quantification is shown in right panels.

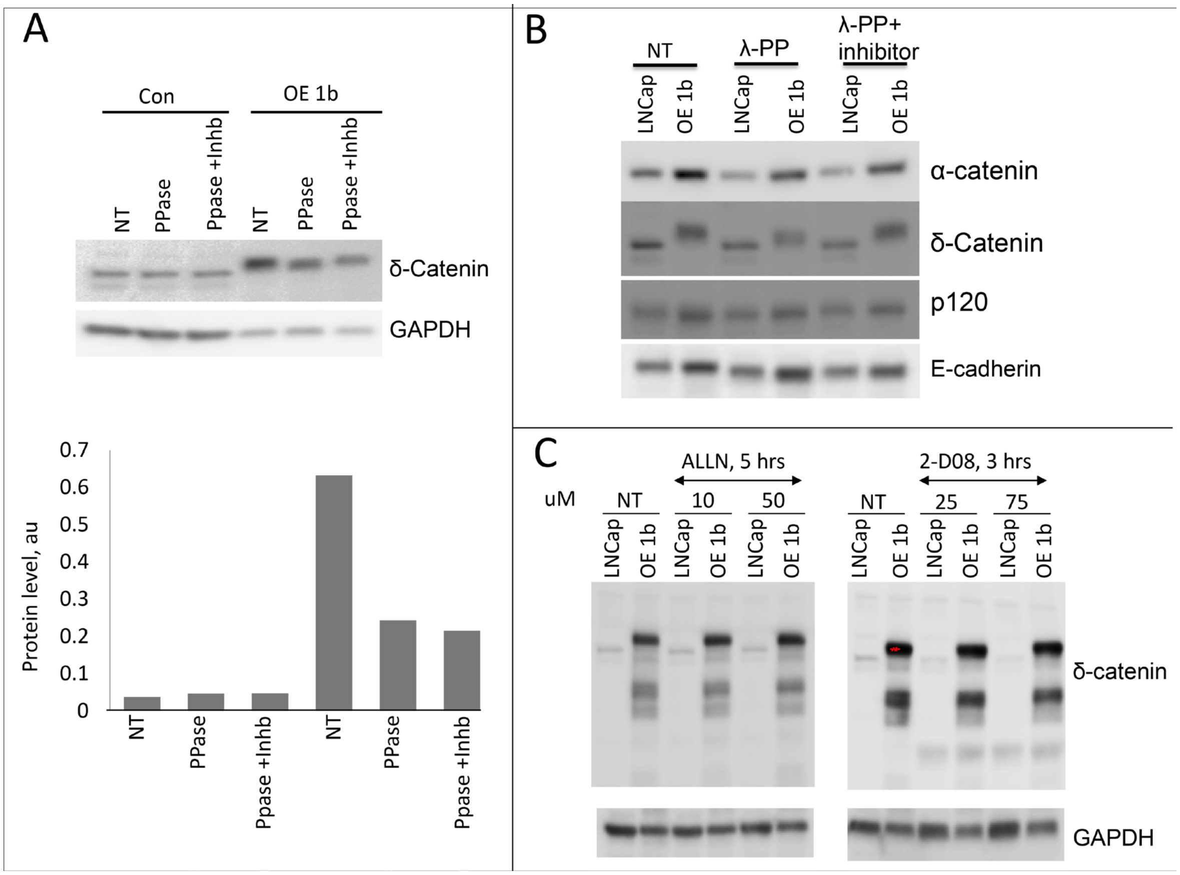

Supplemenatry Figure 4: Examination of posttranslational modifications of catenin proteins. (**A**) Parental LNCaP cells and OE1b clone overexpressing δ-catenin were treated with phosphatase (PPase) or PPase + phosphatase inhibitor for 30 min at 37° C, and levels of δ-catenin were analyzed by Western blotting. Quantification is shown at the bottom. (**B**) Levels of indicated catenins and E-cadherin were compared in LNCap parental cells and OE1b clone before and after treatment with PPase or PPase+inhibitor. Treatments were conducted as in A. (**C**) Levels of δ-catenin in LNCaP cells and OE1b clone were compared before and after treatment with proteosome inhibitor ALLN (left panel) or 2-D08, inhibitor of sumoylation (right panel). Time and concentrations are as indicated.

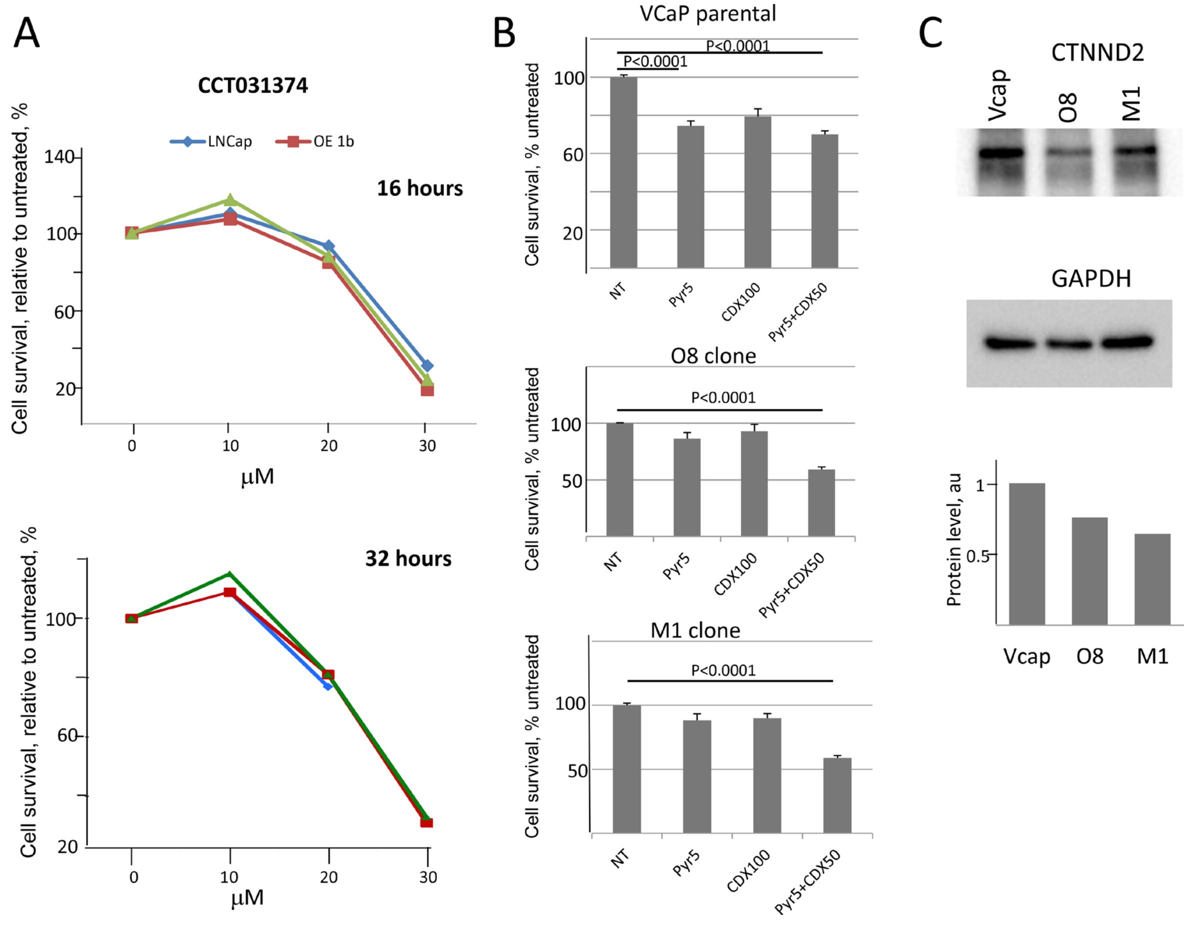

Supplementary Figure 5: (**A**) Sensitivity of parental LNCaP cells and δ-catenin overexpressing clones OE1b and OE3a to treatments with β-catenin inhibitor CCT031374. Concentrations and time are as indicated. (**B**) Sensitivity of parental VCaP cells and their derivative clones O8 and M1 to indicated treatments. Cells were grown in RPMI 1640 supplemented with 10% charcoal stripped FBS for 48 hours and then treated with pyrvinium (Pyr) or casodex (CXD) or their combination for 18 hours. Concentrations and as indicated. Data are presented as mean ± SD. (**C**) Western blot analysis of δ-catenin levels to confirm reduction in O8 and M1 clones. 30 µg of cell lysates were used, representative gel and corresponding quantification are shown.
